# Supplementary material for: Cell state dependent effects of Bmal1 on melanoma immunity and tumorigenicity
Source: Nat Commun. 2024 Jan 20;15:633. doi: 10.1038/s41467-024-44778-2 (PMC10799901; doi:10.1038/s41467-024-44778-2)
Supplement: Supplementary file 1 — Supplementary Information [file 41467_2024_44778_MOESM1_ESM.pdf]

Cell State Dependent Effects of Bmal1 on Melanoma Immunity and Tumorigenicity

Xue Zhang, Shishir M Pant, Cecily C. Ritch, Hsin-Yao Tang, Hongguang Shao, Harsh Dweep, Yao-Yu Gong, Rebekah Brooks, Patricia Brafford, Adam J. Wolpaw, Yool Lee, Ashani Weeraratna, Amita Sehgal, Meenhard Herlyn, Andrew Kossenkov, David Speicher, Peter K. Sorger, Sandro Santagata, Chi V. Dang

Supplementary Information includes eight Supplementary Figures and one Supplementary Table

Supplementary Fig.1 Loss of Bmal1 Decreases YUMM2.1 Tumorigenesis.

Supplementary Fig.2 Ectopic Expression of Bmal1 Affects Immune Infiltration and Accelerates Tumorigenesis of YUMM2.1.

Supplementary Fig.3 Ectopic Expression of Bmal1 Induces Sox10^{high} YUMM2.1 Cells toward Sox9^{high} More Mesenchymal Cell State

Supplementary Fig.4 Bmal1 Interacts with Myh9 in Nucleus

Supplementary Fig.5 Bmal1 and Myh9 Interaction Increases MRTF-SRF Activity and Drives Cell State Change.

Supplementary Fig.6 Myh9 Knockdown Drives YUMM2.1 Cells to More Mesenchymal Cell State.

Supplementary Fig.7 Loss of Myh9 Affects Immune infiltration and Increases Tumorigenesis of YUMM2.1.

Supplementary Fig.8 All uncropped immunoblot scans presented in Supplementary Figures

Supplementary Table 1. Information about reagents and resources

Supplementary Figure. 1

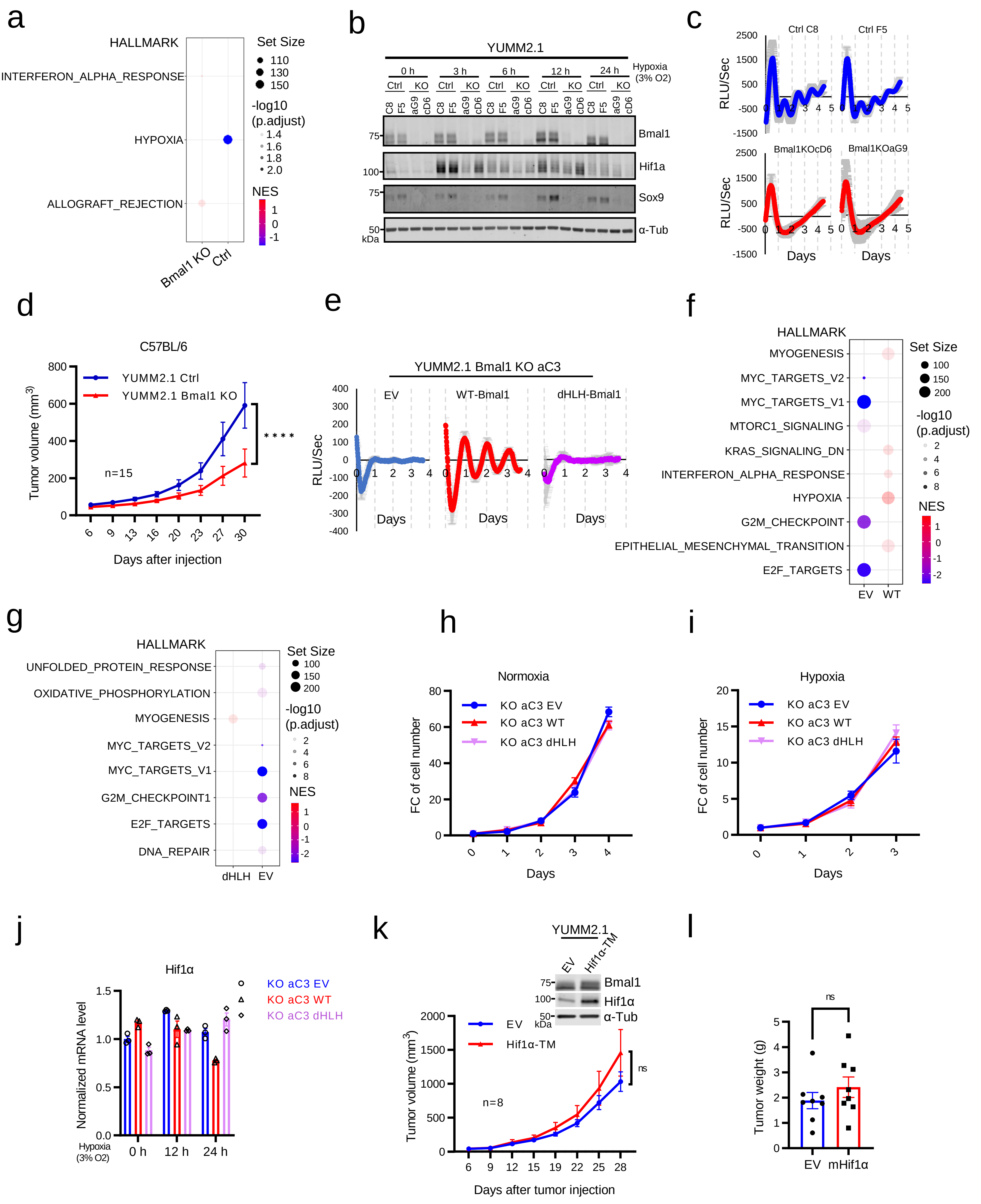

Supplementary Fig. 1

a, GSEA showing all gene sets that are enriched in Bmal1 KO cells (positive NES) or Ctrl cells (negative NES) from duplicate Quant-seq. **b,** Immunoblot for Bmal1, Hif1α and Sox9 protein levels in YUMM2.1 control clones (C8 and F5) and Bmal1 KO clones (aG9 and cD6). RE of 2. **c,** Real-time luminescence monitoring of *Bmal1*::dLUC in YUMM2.1 control clones (C8, F5) and Bmal1-null clones (aG9 and cD6) synchronized with dexamethasone. Luminescence signal is baseline subtracted and data are shown starting 24 hours after synchronization. Signal confidence interval is presented in gray. Mean ± SEM of 3 BR, RE of 2. **d**, Tumor growth rate of control clones (C8, F5) and Bmal1 KO clones (aG9 and cD6) in C57BL/6 mice (n=15 each group). ****p-value <0.0001 by Two-way ANOVA test. **e,f,** GSEA showing all gene sets enriched in Bmal1 KO aC3 WT-Bmal1 (positive NES) vs EV cells (**e**) or dHLH-Bmal1 (positive NES) vs EV cells (**f**) from duplicate Quan-seq. **g**, Real-time luminescence monitoring of *Bmal1*::dLUC in aC3 with EV, WT-Bmal1 and dHLH-Bmal1 synchronized with dexamethasone. Luminescence signal is baseline subtracted and data are shown starting 28 hours after synchronization. Mean ± SEM of 3 BR, RE of 2. **h,i,** Cell growth rate under normoxia (**h**) and 3% O_2 hypoxia (**i**). Mean ± SEM of 3 BR. **j**, Hif1α mRNA level from aC3 with EV, WT-Bmal1 and dHLH-Bmal1 at different time points after exposure to 3% O_2. Mean ± SEM of 3 BR. **k,l,** Tumor growth rate (**k**) and tumor weight (**l**) of YUMM2.1 EV and YUMM2.1 Hif1α-TM cells in C57BL/6 mice (n=8 each group). p-value by Two-way ANOVA test in **k**, Two-tailed unpaired t test in **l**. BR = biological replicate; RE = replicate experiment.

Supplementary Figure. 2

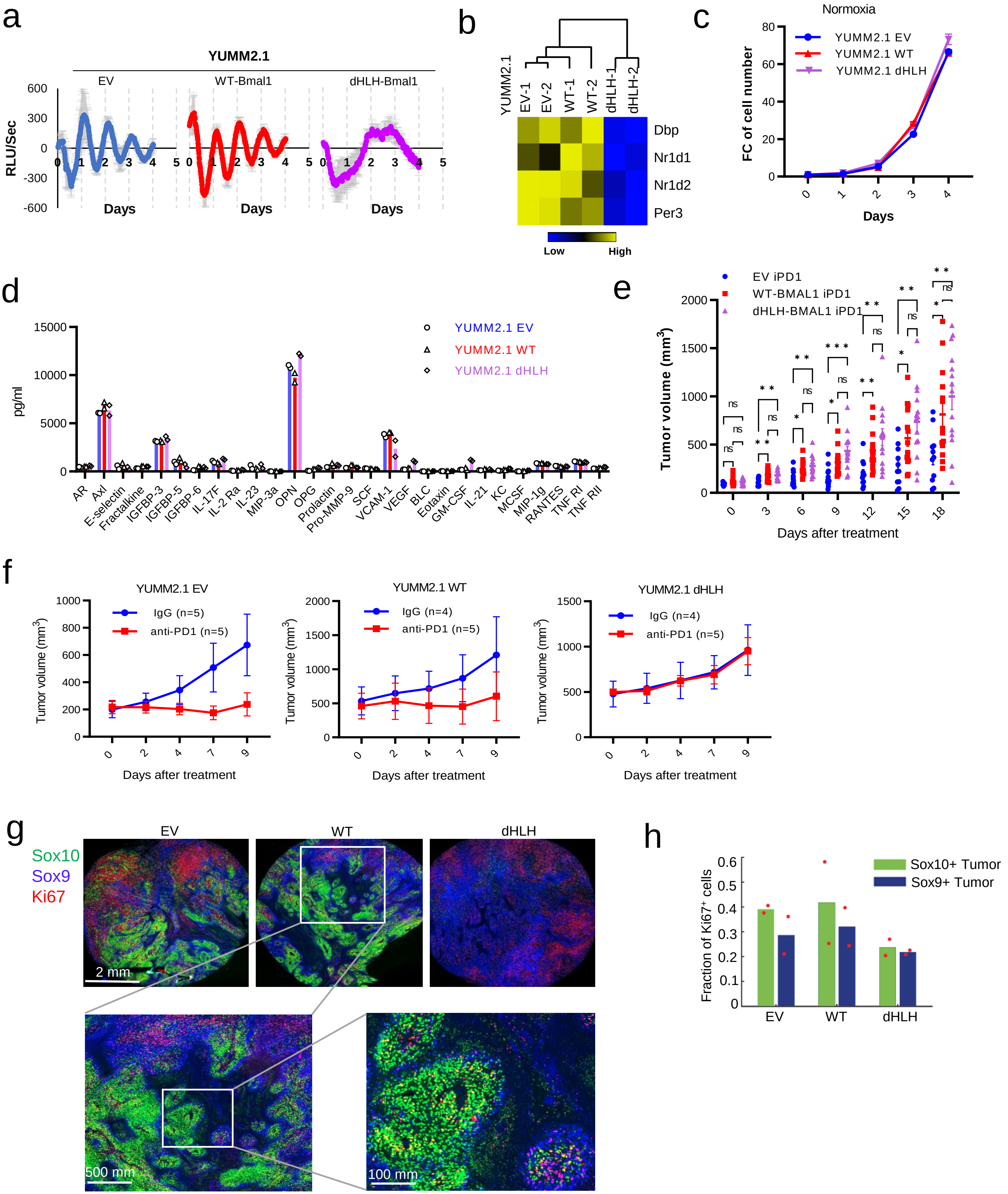

Supplementary Figure. 2

i

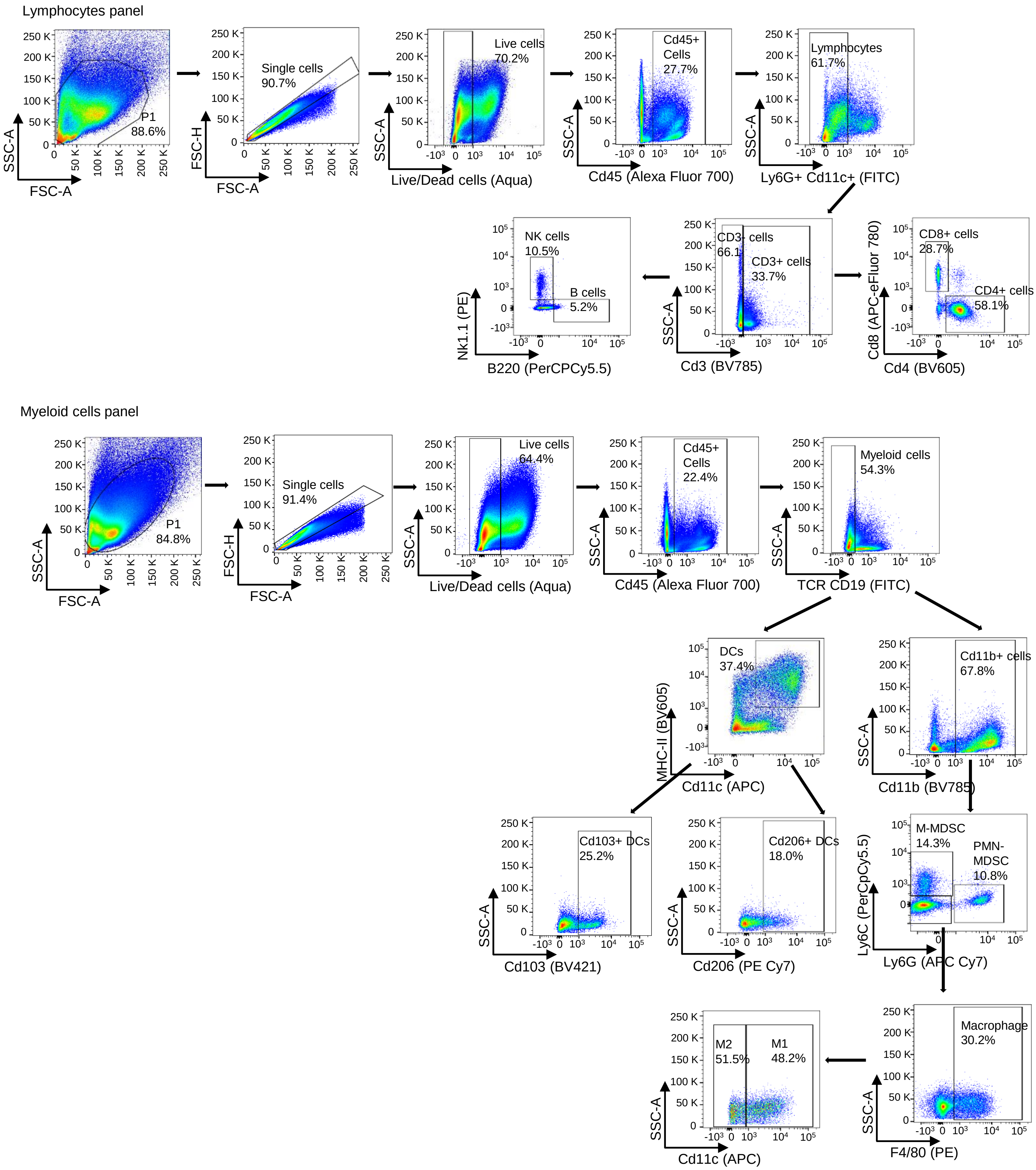

Supplementary Fig. 2

a, Detrended real-time luminescence monitoring of *Arntl*::dLUC in YUMM2.1 with EV, WT-Bmal1 and dHLH-Bmal1 synchronized with dexamethasone for up to 4 days. Confidence intervals are shown in gray. BR of 3. RE of 2. **b,** Heatmap of Bmal1 direct target genes from Quant-seq of YUMM2.1 EV, WT-Bmal1 and dHLH-Bmal1 cells in biological replicates. **c,** In vitro cell growth rates of YUMM2.1 EV, WT-Bmal1 and dHLH-Bmal1 under normoxia. Mean ± SD of 3 BR. RE of 3. **d,** Cytokines released from YUMM2.1 EV, WT-Bmal1 and dHLH-Bmal1 cells in vitro. BR of 2. Each dot represents one biologically independent sample. **e,** Individual volume of tumors in **Fig. 2f** with anti-PD1 treatment given IP every three days. ns: no significance, *p-value <0.05, ** p-value <0.01, ***p-value <0.001 by Two-way ANOVA test followed by Tukey's multiple comparisons test. **f,** Earlier replicate experiment of response of YUMM2.1 EV, WT-Bmal1 and dHLH-Bmal1 tumors in female C57BL/6 mice to anti-PD1 (iPD1) or IgG control treatment. WT-Bmal1: IgG (n=4), iPD1 (n=5); dHLH-Bmal1: IgG (n=4), iPD1 (n=5). **g,** Cyclic immunofluorescent microscopy (CyCIF) for Ki67 (red), Sox9 (blue) and Sox10 (green) in YUMM2.1 EV, WT-Bmal1 and dHLH-Bmal1 tumor tissues from C57BL/c mice. **h,** Percentage of Ki67+ melanoma cells (Sox9+ or Sox10+ cells) in tumor tissues determined by CyCIF. Each dot represents one tissue from two independent tumor tissues (n=2). **i,** Gating strategy of immunophenotyping for tumor tissues in C57BL6/c mice. BR = biological replicate; RE = replicate experiment.

Supplementary Figure. 3

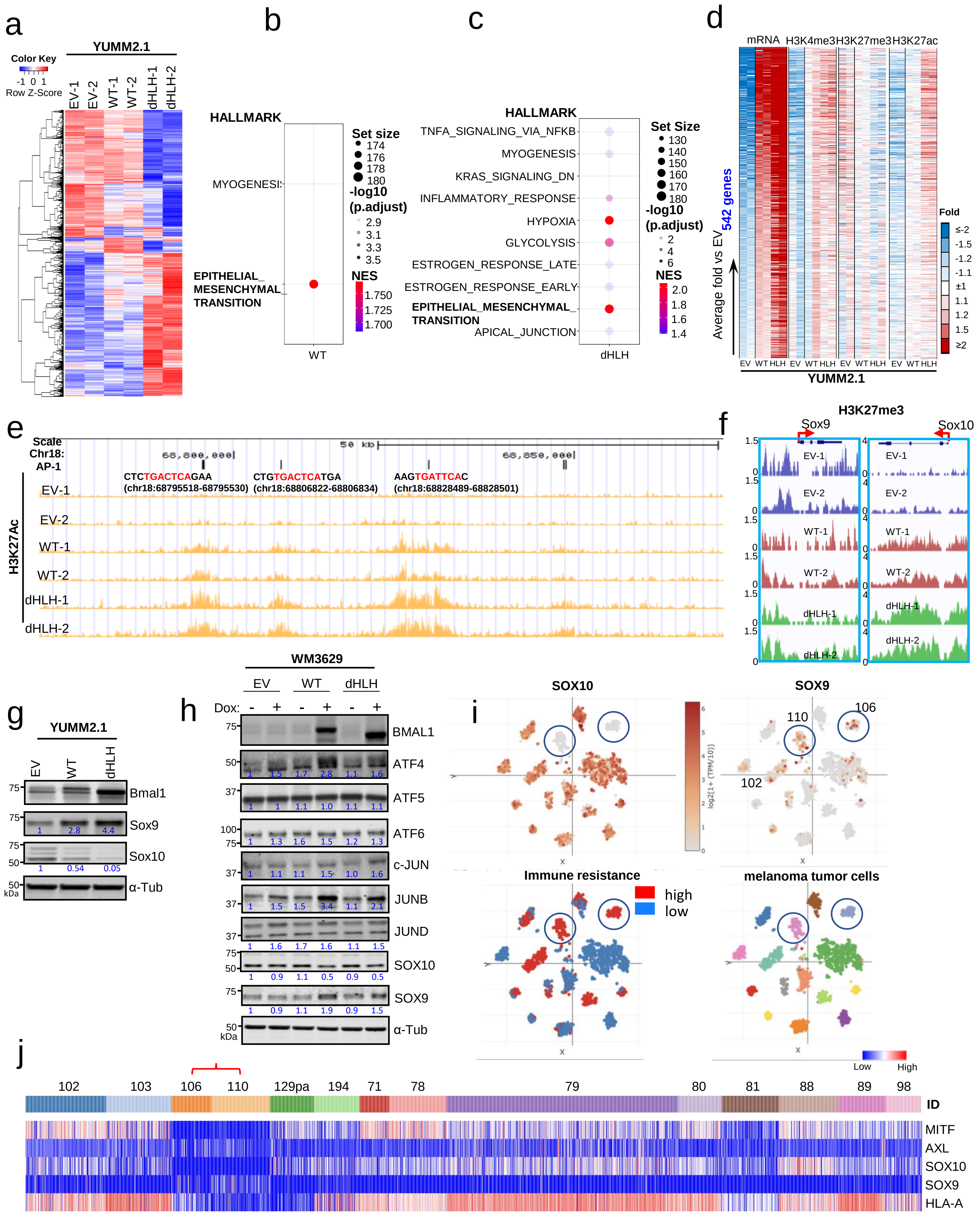

Supplementary Fig. 3

a, Heatmap for all differentially expressed genes in YUMM2.1 EV, WT-Bmal1 and dHLH-Bmal1 cells from biological replicates. **b,c,** GSEA showing all gene sets enriched in YUMM2.1 WT-Bmal1 (positive NES) vs EV cells (**b**) or YUMM2.1 dHLH-Bmal1 (positive NES) vs EV cells (**c**). **d,** Heatmap for mRNA level, H3K4me3 (± 1kb from TSS), H3K27Ac (± 1kb from TSS) and H3K27me3 (in gene body) alterations among 542 genes shown in Fig. 3a. **e,** A representative intergenic locus with increased H3K27Ac signals in YUMM2.1 WT-Bmal1 and dHLH-Bmal1 cells versus EV cells. AP-1 consensus motifs were illustrated on the top. Samples are in biological duplicates. **f,** H3K27me3 alterations at Sox9 and Sox10 loci among YUMM2.1 EV, WT-Bmal1 and dHLH-Bmal1 cells from duplicate CHIP-Seq data. **g,** Immunoblot for Bmal1, Sox9 and Sox10 in YUMM2.1 EV, YUMM2.1 WT-Bmal1 and YUMM2.1 dHLH-Bmal1 cells. RE of 4. **h,** Immunoblot for BMAL1, AP-1 factors, SOX9 and SOX10 in human melanoma cell line WM3629 with or without doxycycline induced EV, WT-Bmal1, dHLH-Bmal1. RE of 2. Numbers underneath the rows represent relative expression of proteins on different gels but from the same experiment. **i,** scRNAseq UMAPs for melanoma samples from the Broad Institute Single Cell Portal highlighting expression of SOX10 and SOX9. Immune resistance score is shown for different clusters of melanoma cells. Note clusters of cells without SOX10 expression have increased SOX9 expression and are associated with high immune resistanc[1]. **j,** Expression of MITF, AXL, SOX10, SOX9, and HLA-A from tumors in f. Numbers at the top representing patient samples, and heatmap at the bottom showing expression level of 4 genes in each cell across all these tumors. The bracket highlights tumors 106 and 110. RE = replicate experiment.

Supplementary Figure. 4

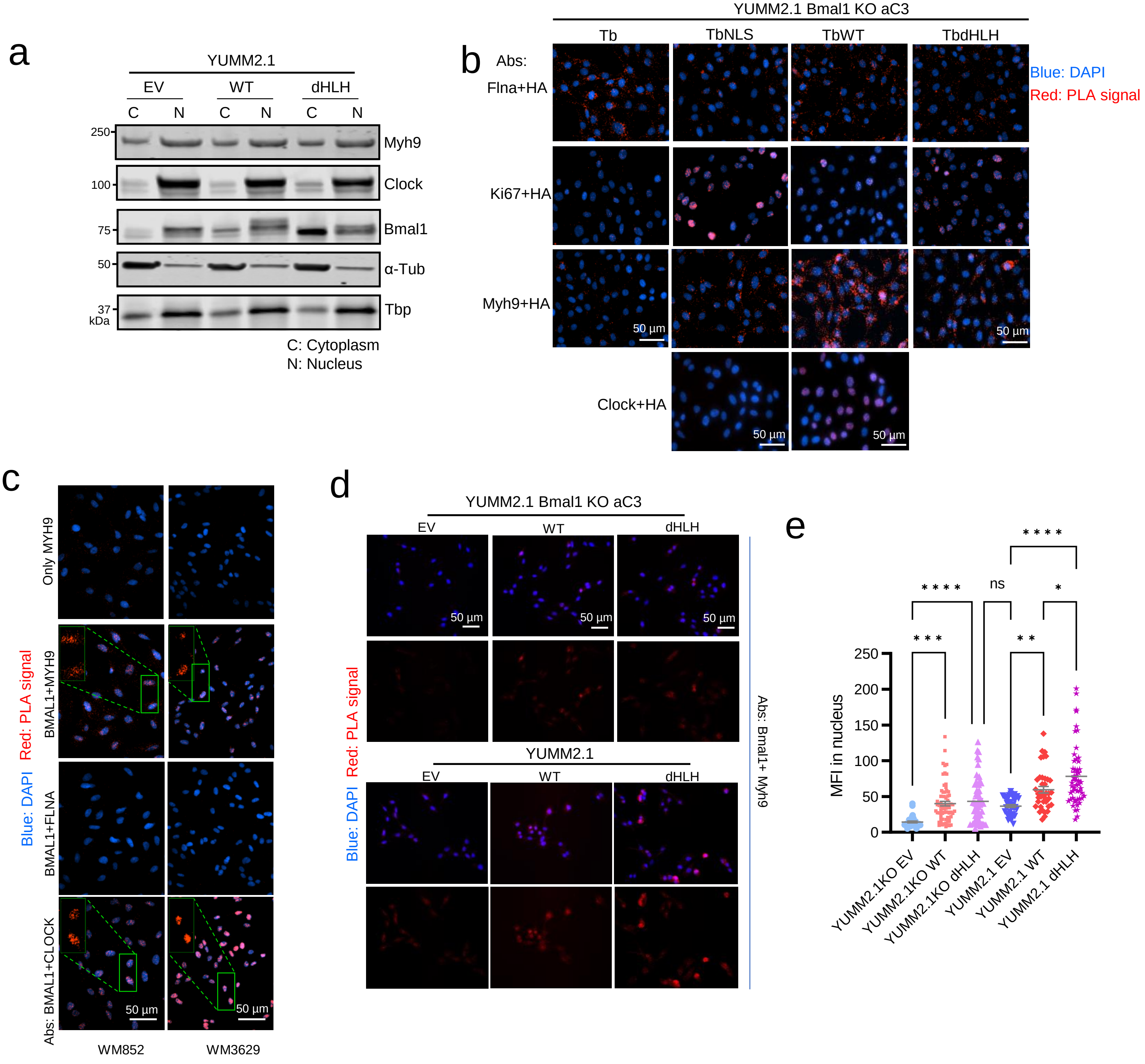

Supplementary Fig. 4

a, Nuclear (N) versus cytoplasmic (C) distribution of Myh9, Clock, Bmal1 in YUMM2.1 EV, WT-Bmal1 and dHLH-Bmal1 cells with α-tubulin and TATA binding protein (Tbp) serving as cytoplasmic and nuclear protein controls, respectively. RE of 3. **b,** Proximity labeling assay controls with filamin A (Flna), Ki67 and Clock as compared with Myh9 across Bmal1 KO aC3 cells expressing Tb, TbNLS, TbWT, and TbdHLH. RE of 2. **c,** PLA showing the interaction between endogenous BMAL1 and MYH9 in human melanoma cell lines WM852 and WM3629. Antibodies against CLOCK and FLNA were used as positive and negative control, separately. Cells in green rectangles were magnified without DAPI signal. RE of 2. **d,** PLA showing Bmal1 and Myh9 in situ interaction in YUMM2.1 Bmal1 KO aC3 EV, WT-Bmal1, dHLH-Bmal1, YUMM2.1 EV, WT-Bmal1 and dHLH-Bmal1 cells. RE of 2. **e,** MFI of nuclear PLA signals in YUMM2.1 Bmal1 KO aC3 EV, WT-Bmal1, dHLH-Bmal1, YUMM2.1 EV, WT-Bmal1 and dHLH-Bmal1 cells. *p-value <0.05, ** p-value <0.01, ***p-value <0.001, ****p-value < 0.0001 by one-way ANOVA test followed by multiple comparison test. RE of 2. RE = replicate experiment.

Supplementary Fig. 5

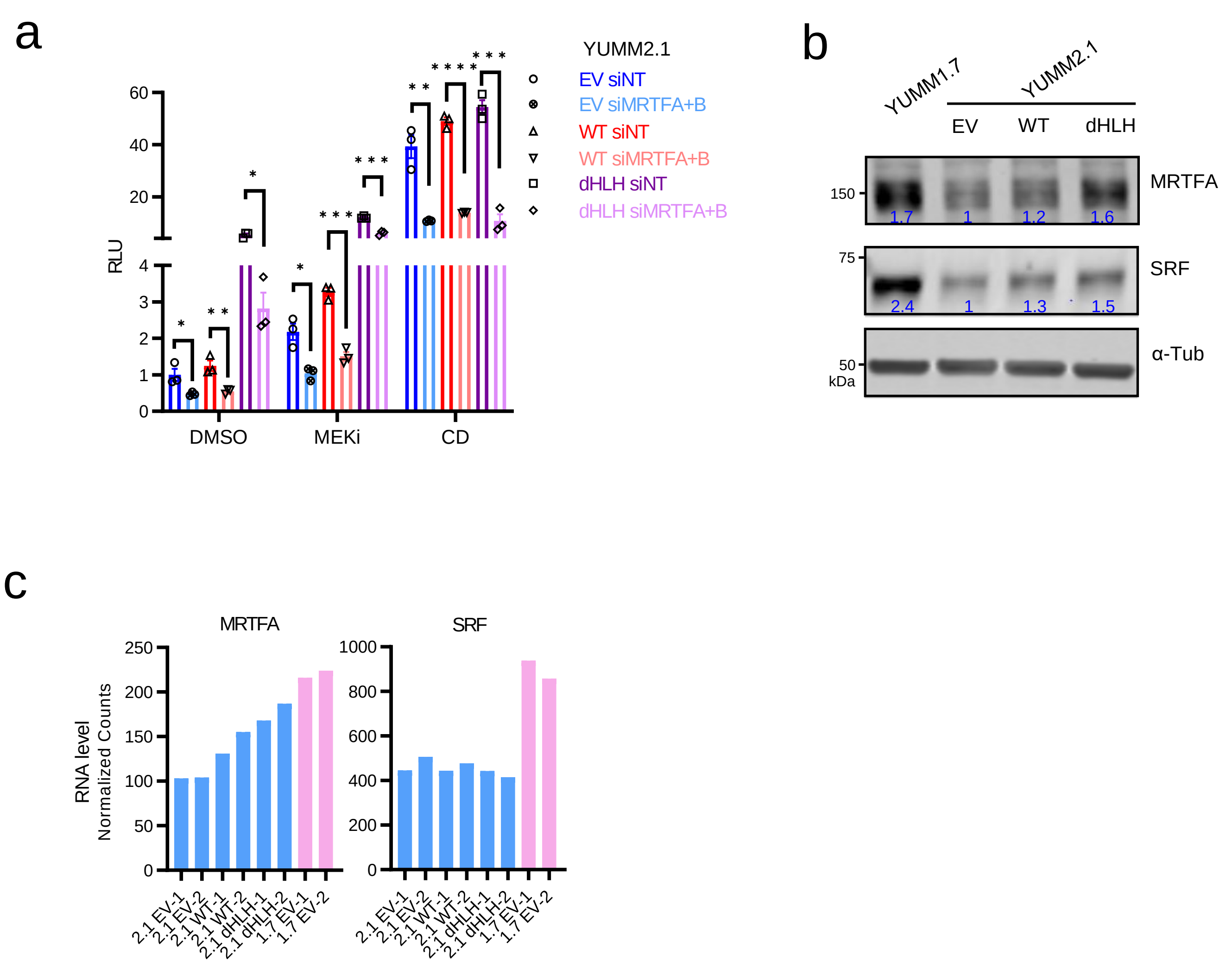

Supplementary Fig. 5

a, Relative luminescence (RLU) from cells YUMM2.1 EV, WT-BMAL1 and dHLH-BMAL1 treated with siRNAs targeting MRTFA and MRTFB followed by transient transfection with SRF-RE luciferase and Renilla luciferase plasmids. Cells were then treated with 10 nM Trametinib (MEKi) or 2 μM Cytochalasin D (CD) for 20 hours and 2 hours, respectively. DMSO was a control for 20 hours treatment. Mean ± SEM of 3 BR. RE of 2. *p-value <0.05, **p-value <0.01, ***p-value <0.001, ****p-value <0.0001 by one-way ANOVA followed by multiple comparison test. **b,** Immunoblot of MRTFA and SRF in YUMM1.7, YUMM2.1 EV, WT-Bmal1 and dHLH-Bmal1 cells. Numbers underneath the rows represent relative expression. RE of 3. **c,** mRNA level of MRTFA and SRF in YUMM1.7, YUMM2.1 EV, YUMM2.1 WT-Bmal1 and YUMM2.1 dHLH-Bmal1 from Quant-seq data. BR of 2. BR = biological replicate; RE = replicate experiment.

Supplementary Fig. 6

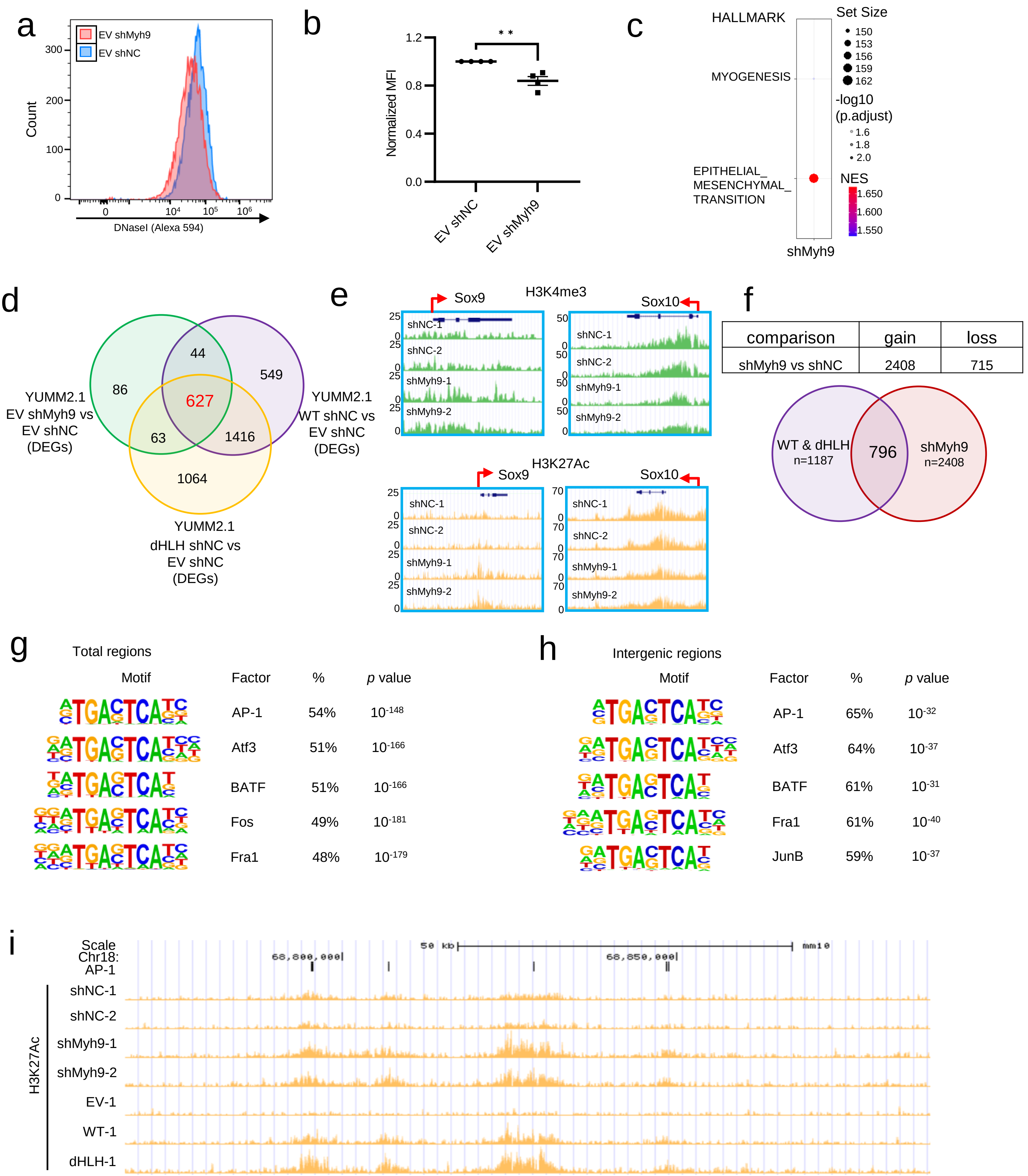

Supplementary Fig. 6

a,b, Flow cytometry analysis of G-actin stained with Alexa594-conjugated DNaseI in YUMM2.1 EV with shNC and shMyh9 cells. Data (**a**) represents 4 independent experiments which were quantified in (**b**). Data was normalized to EV. **p-value <0.01 by two-tailed unpaired t test. **c,** GSEA showing all gene sets that are enriched in YUMM2.1 EV shMyh9 vs shNC. Samples are from biological duplicates. **d,** Venn diagram showing the number of overlapping differentially expressed genes (DEGs) in the same direction from 3 comparisons. Green circle for DEGs from EV shMyh9 vs EV shNC, orange circle for DEGs from WT shNC vs EV shNC and purple circle for DEGs from dHLH shNC vs EV shNC. **e,** H3K4me3 and H3K27Ac alterations at Sox9 and Sox10 loci in YUMM2.1 EV shNC and shMyh9 cells from duplicate CHIP-Seq data. **f,** Gain or loss of H3K27Ac CHIP-seq signals from YUMM2.1 shMyh9 versus shNC. Venn diagram showing the overlapping number of regions gaining H3K27Ac signal between WT/dHLH versus EV and shMyh9 versus shNC. Samples are in duplicates. **g,h,** Prevalence of bZIP (AP-1) motif in all 2408 regions (**g**) and 271 intergenic regions (>20 kb from TSS) (**h**). **i,** The same intergenic locus as shown in Supplementary Fig. 3e displaying increased H3K27Ac signal by shMyh9 versus shNC in YUMM2.1 EV cells (top), similar with the change induced by WT-Bmal1 and dHLH-Bmal1 versus EV (bottom).

Supplementary Fig. 7

a

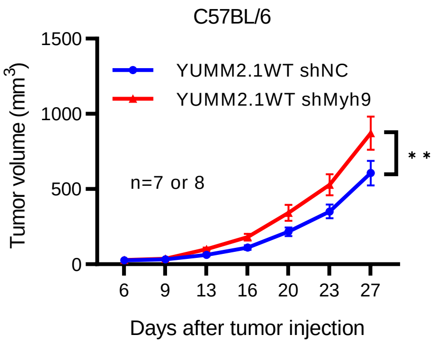

b

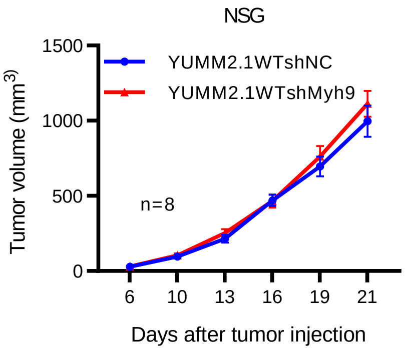

c

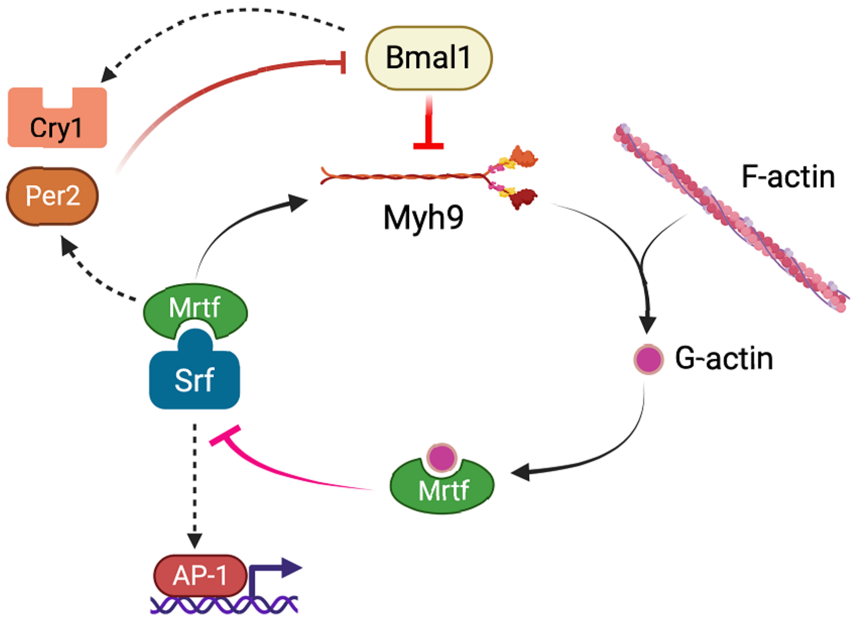

Supplementary Fig. 7

a,b, In vivo tumorigenesis of YUMM2.1 WT with shNC (n=8) or shMyh9 (n=7) in C57BL/6 mice (**a**) and NSG mice (**b**, n= 8 each group). **p-value <0.01 by Two-way ANOVA test. **c,** Cartoon illustrating putative protein-protein interaction (solid lines) and transcriptional (dashed lines) regulatory loops linking MRTF-SRF to the core circadian clock via Bmal1 and AP-1 activation. This model incorporates findings from the literature and our studies. Created with BioRender.com.

Supplementary Fig. 8

All uncropped immunoblot scans presented in Supplementary Figures

Corresponding to Supplementary Fig.1b

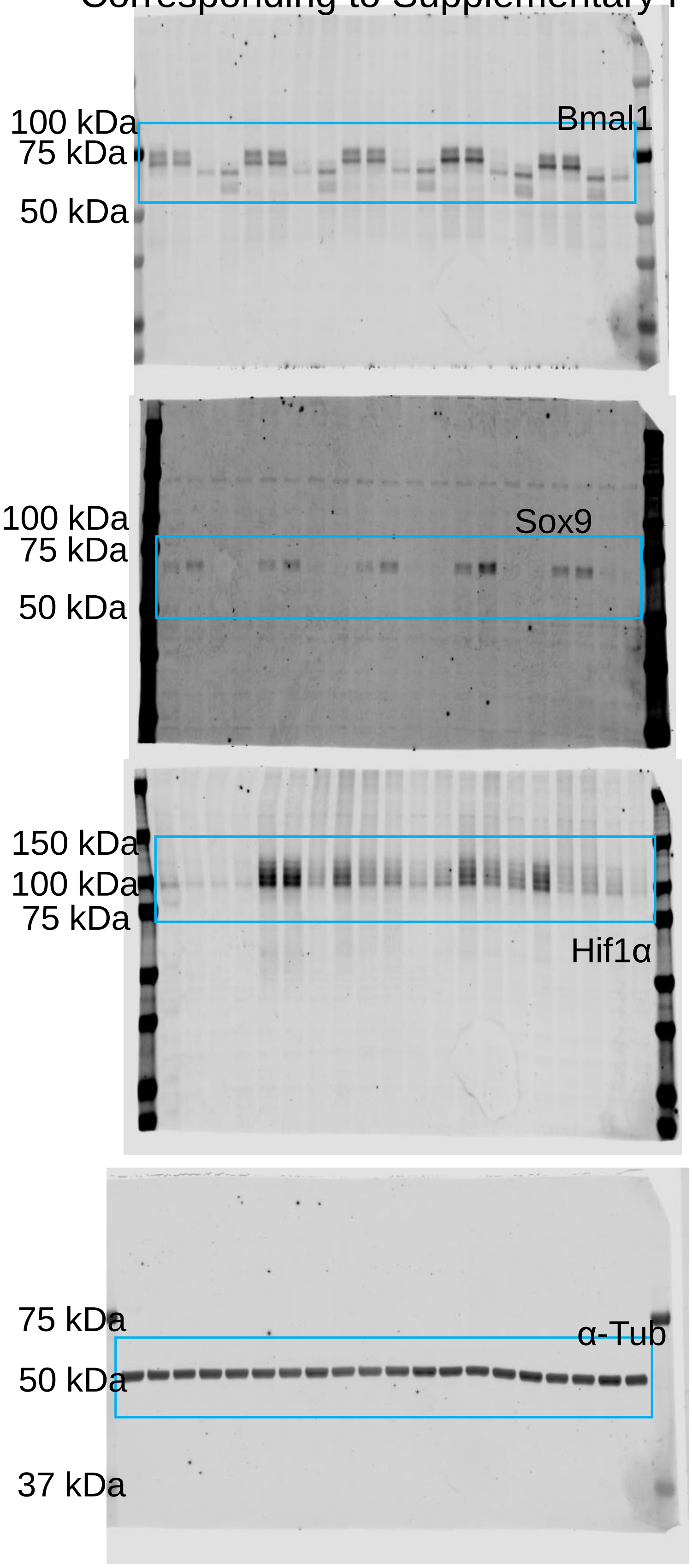

Corresponding to Supplementary Fig. 1k

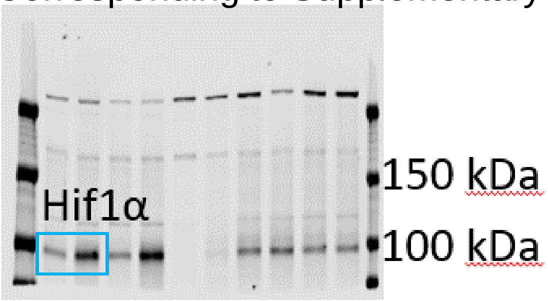

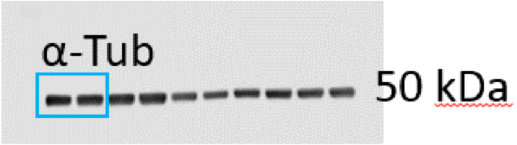

Corresponding to Supplementary Fig. 3g

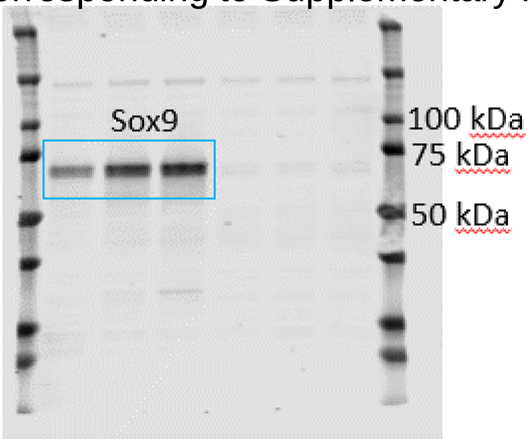

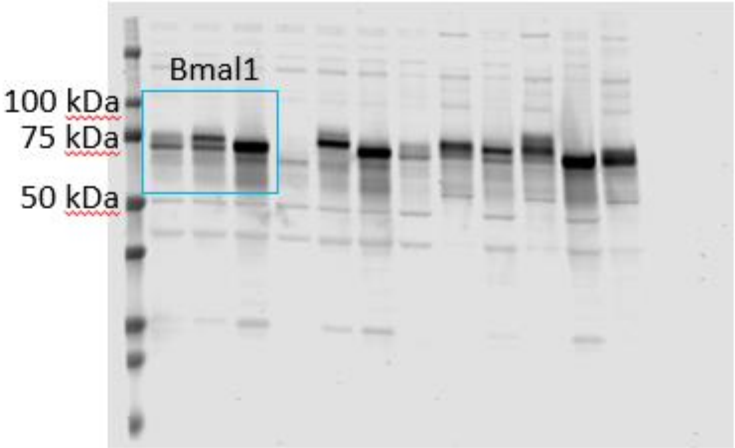

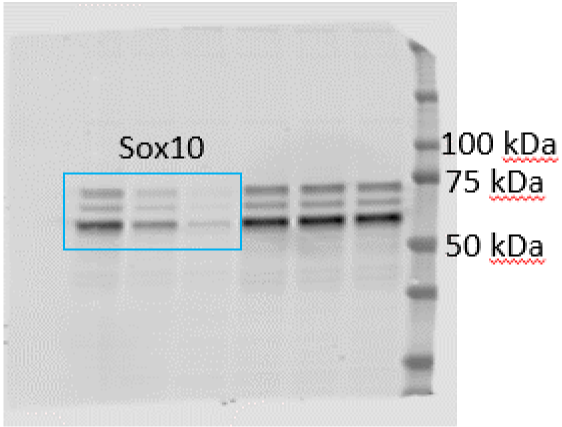

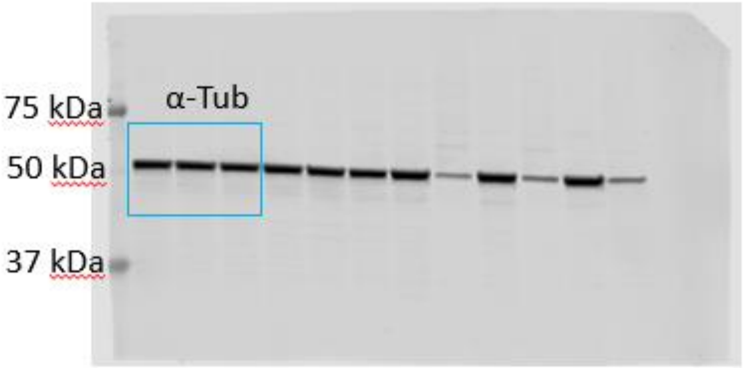

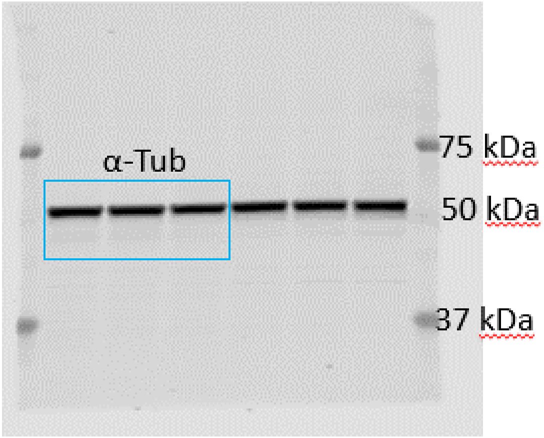

Corresponding to Supplementary Fig.3h

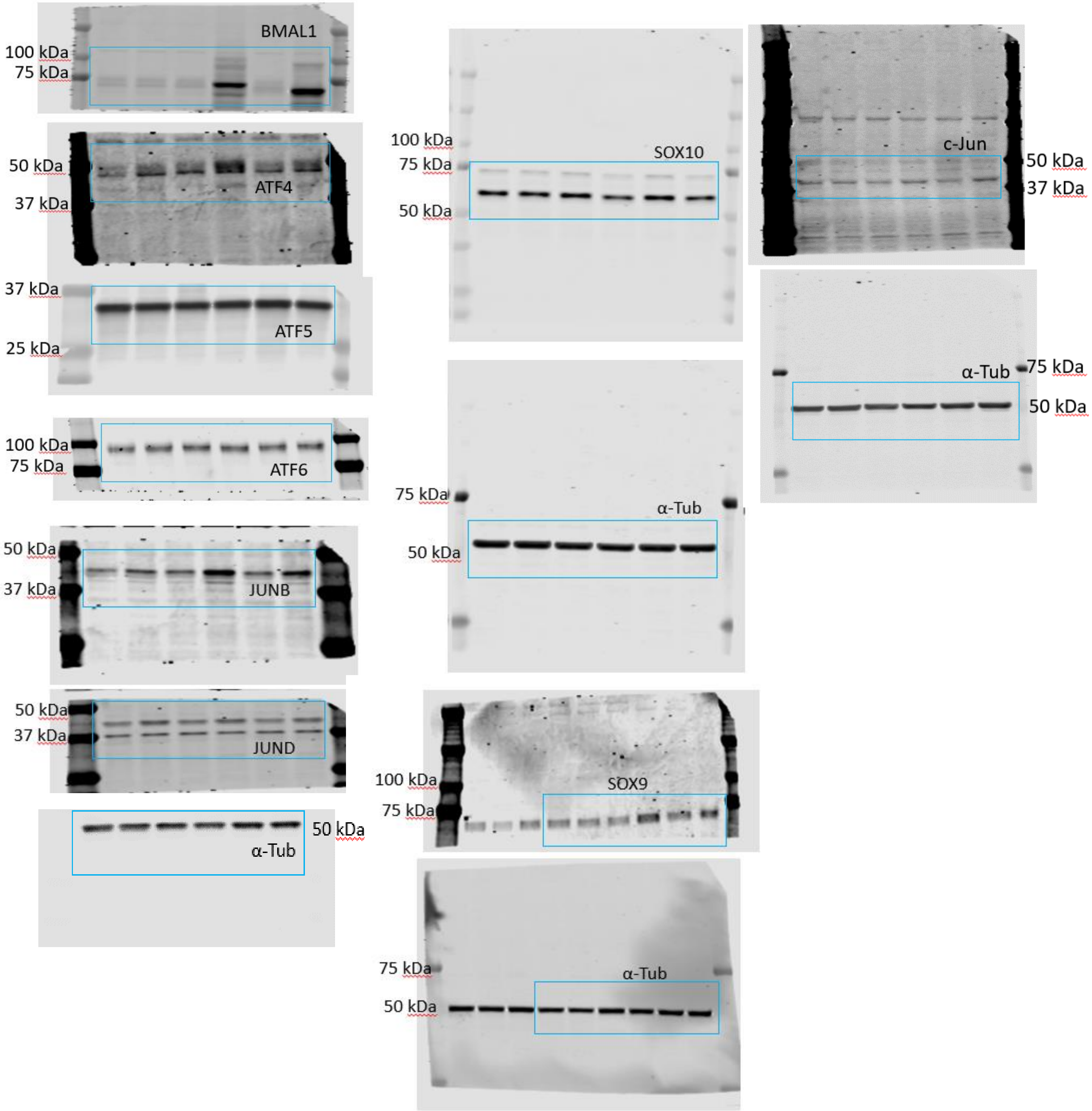

Corresponding to Supplementary Fig.4a

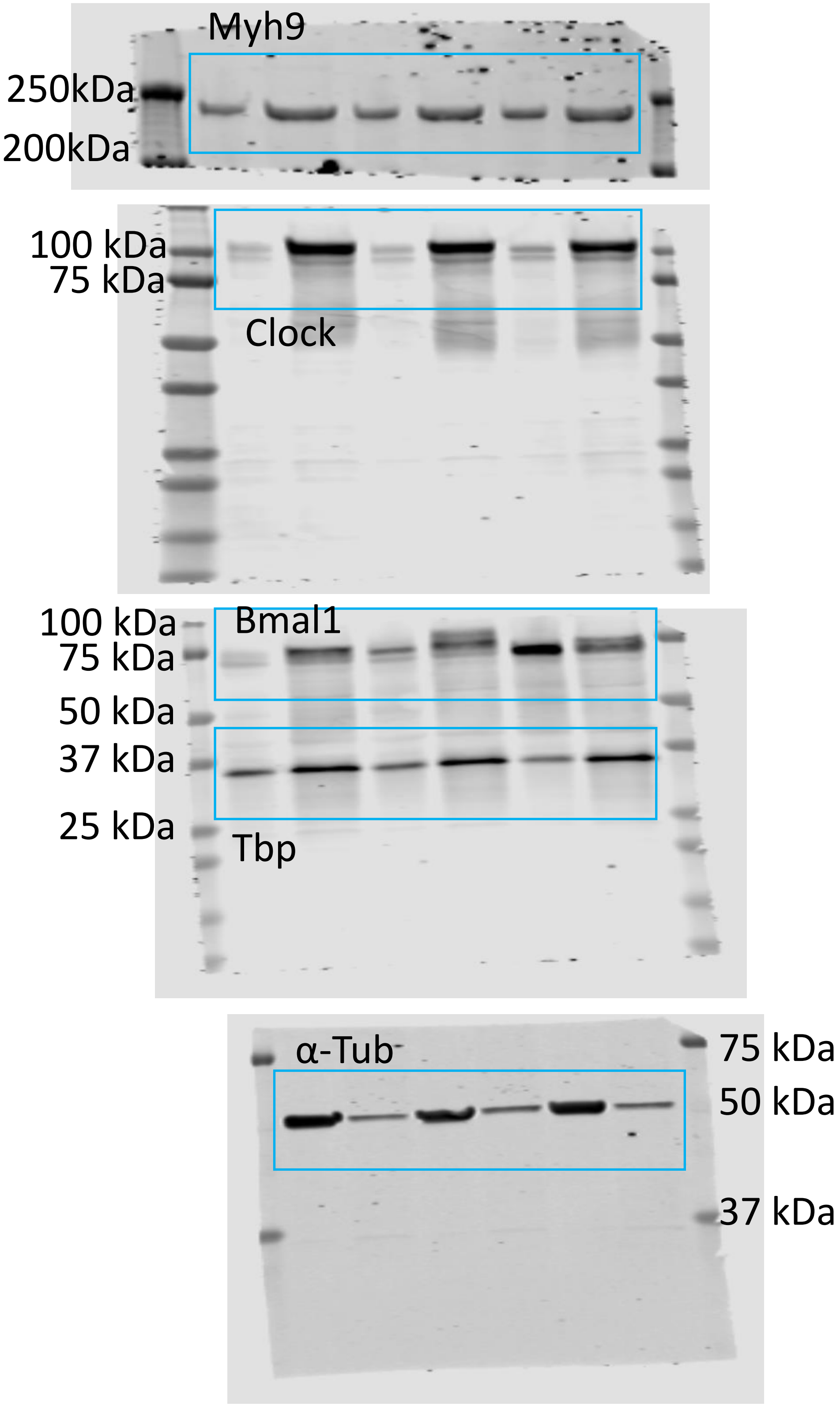

Corresponding to Supplementary Fig. 5b

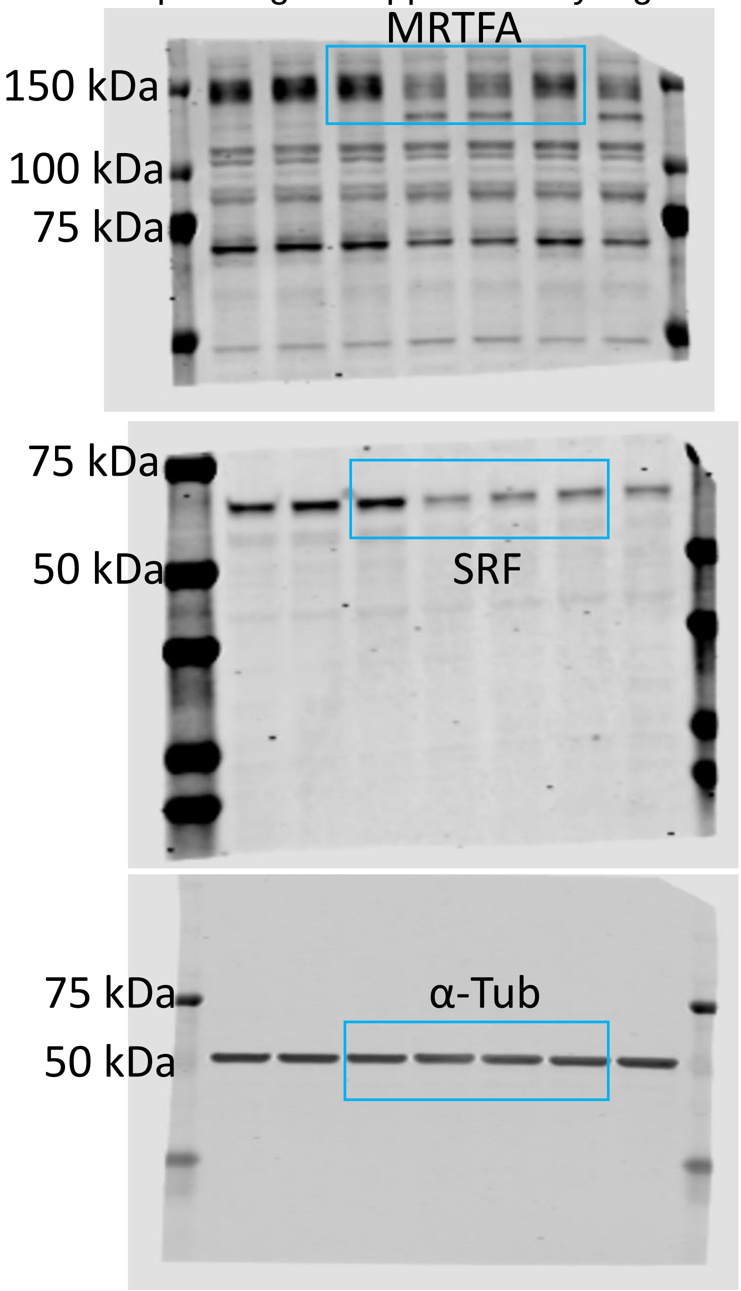

Supplementary Fig.8

All uncropped immunoblot scans corresponding to the immunoblot data presented in Supplementary Figures.

Supplementary Table 1

Reagent or Resource	Source	IDENTIFIER
**Antibodies**		
**Anti-BMAL1 (D2L7G)**	Cell Signaling	Cat#: 14020
**Anti-SOX9 (D8G8H)**	Cell Signaling	Cat#: 82630
**Anti-SOX10 (D5V9L)**	Cell Signaling	Cat#: 89356
**Anti-HIF1α (D1S7W)**	Cell Signaling	Cat#: 36169
**Anti-CLOCK (D45B10)**	Cell Signaling	Cat#: 5157
**Anti-ATF4 (D4B8)**	Cell Signaling	Cat#: 11815
**Anti-CHOP (L63F7)**	Cell Signaling	Cat#: 2895
**Anti-MiTF (D5G7V)**	Cell Signaling	Cat#: 12590
**Anti-Ki67 (D3B5)**	Cell Signaling	Cat#: 9129
**Anti-H3K4me3 (C42D8)**	Cell Signaling	Cat#: 9751
**Anti-H3K27me3 (C36B11)**	Cell Signaling	Cat#: 9733
**Anti-H3K27Ac**	Cell signaling	Cat#: 8173
**Anti-Actn4 (D7U5A)**	Cell Signaling	Cat#: 15145
**Normal Rabbit IgG**	Cell Signaling	Cat#: 2729
**Anti-MLK1**	Millipore Sigma	Cat#: HPA030782
**Anti-MYH9**	Proteintech	Cat#: 11128-1-AP
**Anti-c-Myc (Y69)**	Abcam	Cat#: ab32072
**Anti-Filamin A (EP2405Y)**	Abcam	Cat#: ab76289
**Anti-HA (12CA5)**	Millipore Sigma	Cat#: 11583816001
**Anti-EZH2**	Active Motif	Cat#: 39933

**Anti-α-Tubulin Mouse Mab (DM1A)**	Millipore Sigma	Cat#: CP06
**Anti-BMAL1 (B-1)**	Santa Cruz	Cat#: sc365645
**Anti-TBP**	Cell Signaling	Cat#: 44059
**Anti-c-Jun**	Cell Signaling	Cat#: 9165
**Anti-phospho-c-Jun (ser73)**	Cell Signaling	Cat#: 3270
**Anti-Junb**	Cell Signaling	Cat#: 3753
**Anti-Jund**	Cell Signaling	Cat#: 5000
**Anti-Atf5**	Millipore Sigma	Cat#: ABE2614
**Anti-Atf6**	Cell Signaling	Cat#: 65880
**Anti-Flag**	Cell Signaling	Cat#: 14793
**Anti-Flag M2**	Millipore Sigma	Cat#: F1804
**InVivoMab anti-mouse PD1 (CD279) (RMP1-14)**	BioXCell	Cat#: BE0146
**InVivoMAb rat IgG2a isotype control (2A3)**	BioXcell	Cat#: BE0089
**Goat anti-Rabbit IgG (H+L) highly Crossed-Adsorbed Secondary Ab, Alexa Fluor 680**	Invitrogen	Cat#: A-21109
**Anti-mouse IgG(H+L) (DyLight 800 4X PEG conjugate)**	Cell Signaling	Cat#: 5257
**FITC anti-mouse TCR β chain (H57-597)**	Biolegend	Cat#: 109205
**FITC anti-mouse CD19 (6D5)**	Biolegend	Cat#: 115505

**Brilliant Violet 785™ anti-mouse/human CD11b (M1/70)**	Biolegend	Cat#: 101243
**APC/Cyanine7 anti-mouse Ly-6G (1A8)**	Biolegend	Cat#: 127623
**PerCP-Cy™ 5.5 Rat Anti-Mouse Ly-6C (AL-21)**	BD Biosciences	Cat#: 560525
**PE Rat Anti-Mouse F4/80 (BM8)**	eBioscience	Cat#: 12-4801-80
**APC Hamster Anti-Mouse CD11c (HL3)**	BD Biosciences	Cat#: 561119
**Brilliant Violet 605™ anti-mouse I-A/I-E (M5/114.15.2)**	Biolegend	Cat#: 107639
**Brilliant Violet 421™ anti-mouse CD103 (2E7)**	Biolegend	Cat# : 121421
**PE/Cyanine7 anti-mouse CD206 (C068C2)**	Biolegend	Cat# : 141720
**Alexa Fluor 700 anti-mouse CD45 (30-F11)**	Biolegend	Cat#: 103128
**FITC anti-mouse Ly-6G (1A8)**	Biolegend	Cat#: 127605
**FITC Hamster Anti-Mouse CD11c (HL3)**	BD Biosciences	Cat#: 557400
**Brilliant Violet 785™ anti-mouse CD3ε (145-2C11)**	Biolegend	Cat# : 100355

**PE Mouse Anti-Mouse NK1.1 (PK136)**	BD Biosciences	Cat#: 557391
**PerCP-Cy5.5 Rat Anti-Mouse CD45R/B220 (RA3-6B2)**	BD Biosciences	Cat#: 561101
**APC-Cy™7 Rat Anti-Mouse CD8α (53-6.7)**	BD Biosciences	Cat#: 557654
**Brilliant Violet 605™ anti-Mouse CD4 (GK1.5)**	Biolegend	Cat# : 100451
**Purified Rat Anti-Mouse CD16/CD32 (Mouse BD Fc Block™) (2.4G2)**	BD Biosciences	553142
**Alexa Fluor 555 anti-SOX9 (EPR14335)**	Abcam	Cat#: ab202516
**Alexa Fluor 647 anti-Sox10 (SP267)**	Abcam	Cat#: ab270151
**Goat anti-Rabbit IgG (H+L) highly Crossed-Adsorbed Secondary Ab, Alexa Fluor 647**	Invitrogen	Cat#: A-21245
**Chemicals, peptides, and recombinant proteins**		
**Dabrafenib**	Selleckchem	Cat#: S2807
**Trametinib**	Selleckchem	Cat#: S2673
**Vemurafenib**	Selleckchem	Cat#: S1267
**Cytochalasin D**	Sigma Aldrich	C8273; CAS

		22144-77-0
**Blasticidin S HCl**	Gibco	A1113903
**Puromycin Dihydrochloride**	Gibco	A1113803
**Hygromycin B solution**	Corning	30240CR
**Dexamethasone**	Sigma	D4902; CAS 50-02-2
**Beetle luciferin, potassium salt**	Promega	E1602
**MCDB153 Medium**	Sigma-Aldrich	M7403
**Leibovitz's L-15 Medium**	Gibco	11415064
**Calcium Chloride 2M**	Quality Biological	351-130-721
**DMEM high glucose**	Corning	MT10-013-CV
**Sodium bicarbonate 7.5% solution**	Gibco	25080094
**1M HEPES solution**	Gibco	15630080
**L-Glutamine (200mM) 100X**	Gibco	25030081
**Fetal bovine serum**	HyClone, Gemini Bioproducts	SH30910.03
**Penicillin-Streptomycin Solution (100X)**	Corning	MT30002C1
**High-vacuum silicone grease**	Dow Corning/Sigma	Z273554
**NewBlot ™ Nitro Stripping Buffer**	Li-COR	928-40030
**TRIzol Reagent 200ml**	Invitrogen	15596018
**Formal-Fixx Concentrate**	Thermo Scientific	6764254

**16% Formaldelhyde Solution (w/v), Methanol free**	Thermo Scientific	28908
**Lipofectamine 2000 transfection reagent**	Invitrogen	11668019
**Lipofectamine 3000 transfection reagent**	Invitrogen	L3000015
**Lipofectamine RNAiMAX transfection reagent**	Invitrogen	13778150
**Polybrene Transfection Reagent**	Millipore Sigma	TR-1003-G
**Mammalian protein extraction reagent (M-PER)**	Thermo Scientific	PI78501
**Protease inhibitor cocktail**	Promega	G652A
**Phosphatase inhibitor cocktail 2**	Sigma	P5726
**Phosphatase inhibitor cocktail 3**	Sigma	P0044
**10X RBC Lysis Buffer**	eBioscience	00430054
**2-Mercaptoethanol**	Sigma	M3148; CAS 60-24-2
**DMEM high glucose w/o L-glumatine, phenol red, and sodium bicarbonate (powder)**	USBiological	D9812-05
**Precision Plus protein Dual color standards**	Bio-Rad	1610374

**0.25% Trypsin, 0.1%EDTA**	Corning	25053CI
**Biotin**	Sigma Aldrich	B4639-500mg; CAS 58-85-5
**IRDye 800CW Streptavidin**	LI-COR	926-32230
**Pierce ™ Streptavidin Magnetic Beads**	Thermo Fisher	88816
**10XTBS**	Bio-Rad	1706435
**Bovine Serum Albumin Standard Ampules**	Thermo Fisher	23209
**iBlot™ 2 Transfer Stacks, nitrocellulose**	Invitrogen	IB23001
**Deferoxamine mesylate**	Calbiochem	252750; CAS 138-14-7
**Dynabeads ™ Protein A for Immunoprecipitation**	Invitrogen	10001D
**RIPA Buffer (10X)**	Cell Signaling	9806
**Cell Lysis Buffer (10X)**	Cell Signaling	9803
**Normal Goat Serum**	Cell Signaling	5425
**DNA-free™ Kit DNase Treatment and Removal Reagents**	Invitrogen	AM1906
**Live/Dead Fixable Aqua Dead Cell Stain Kit**	Invitrogen	L34957
**ArC™ Amine Reactive**	Invitrogen	A10628

**Compensation Bead Kit**		
**UltraComp eBeads™ Compensation Beads**	Invitrogen	01-2222-42
**Power SYBR Green PCR Master Mix**	Thermo Fisher	4368708
**TaqMan universal PCR Master Mix**	Thermo Fisher	4304437
**Matrigel Growth Factor Reduced Basement Membrane Matrix, LDEV-free**	Corning	354230
**Critical Commercial Assays**		
**DC protein assay kit II**	Bio-Rad	5000112
**Dual-Luciferase Reporter Assay System**	Promega	E1960
**Tumor Dissociattion Kit (mouse)**	Miltenyi Biotec	130-096-730
**NE-PER Nuclear and Cytoplasmic Extraction Reagents**	Thermo Fisher	78833
**Duolink In Situ Red Starter Kit Mouse/Rabbit**	Millipore Sigma	DUO92101
**In-Fusion HD Cloning Kit**	Takara	639650
**SimpleCHIP Enzymatic Chromatin IP Kit (magnetic Beads)**	Cell Signaling	9003

**FoxP3/Transcription Factor Staining Buffer Set**	Invitrogen	00-5523-00
**CellTiter-Glo Luminescent Cell Viability Assay**	Promega	G7571
**Dead Cell Removal Kit**	Miltenyi Biotec	130-090-101
**Deoxyribonuclease I, Alexa594 Conjuate**	Invitrogen	D12372
**shRNA Target gene**	Sequence	Vector
Myh9	1. CGGTAAATTCATTCGTATCAA<br>2. GCCATACAACAAATACCGCTT	pLKO.1
**Cloning primers**		
**Mhy9 Head Forward**	cccgGCTAGCgccAccatgGACTACAAAGACGATGACGACAAG*GGG*GACTACAAAGACGATGACGACAAG*GGGGGA*gcacagcaagctgccgataagtatctct	
**Myh9 Head Reverse**	ggccGGTACCttacagcagcggcttgaccttggtgaaga	
**Myh9 Tail Forward**	cccgGCTAGCgccAccatgGACTACAAAGACGATGACGACAAG*GGG*GACTACAAAGACGATGACGACAAG*GGGGGA*agccggcaggaggaggagatgatggcca	
**Myh9 Tail Reverse**	ggccGGTACCttattcggcaggtttggcctcagcccca	
**mHif1α Forward**	CGC*TCTAGA*GCCACCatggagggcgccggcggcgagaa	
**mHif1α Reverse**	CGGCGCTAGCtcagttaacttgatccaaagctctgagtaattcttcaccctg	
**ON-TARGETplus Mouse Myh9 siRNA SMARTPool**	Dharmacon	L-040013-00-0005
**ON-TARGETplus Non-targeting Control Pool**	Dharmacon	D-001810-10-05
**ON-TARGETplus Mouse SRF siRNA SMARTPool**	Dharmacon	L-050116-01-0005
**ON-TARGETplus**	Dharmacon	L-054350-00-0005

**MouseMkl1 siRNA SMARTPool**		
**ON-TARGETplus MouseMkl2 siRNA SMARTPool**	Dharmacon	L-054677-00-0005
**Recombinant DNA**		
**psPAX2**	Laboratory of Didier Trono	Addgene plasmid # 12260
**pMD2.G**	Laboratory of Didier Trono	Addgene plasmid # 12259
**pCRISPR-CG01-sgArntl-a**	GeneCopoeia	MCP001575-CG01-3-B-a
**pCRISPR-CG01-sgArntl-b**	GeneCopoeia	MCP001575-CG01-3-B-b
**pCRISPR-CG01-sgArntl-c**	GeneCopoeia	MCP001575-CG01-3-B-c
**pHIG2PW-EV**	This paper	N/A
**pHIG2PW-WT-BMAL1**	This paper	N/A
**pHIG2PW-dHLH-BMAL1**	This paper	N/A
***Arntl*::dLUC reporter**	Laboratory of Andrew Liu	N/A
**pHIG2PW-3XHA-TurboID**	This paper	N/A
**pHIG2PW-3XHA-TurnoID-NLS**	This paper	N/A
**pHIG2PW-3XHA-TurboID-WT-BMAL1**	This paper	N/A
**pHIG2PW-3XHA-TurboID-dHLH-BMAL1**	This paper	N/A
**pGL4.34[*luc2P*/SRF-RE/Hygro]**	Promega	E1350

**Renilla luciferase - Pol III**	Addgene	37380
**pGL 4.73 (Renilla)**	Promega	E6911
**pLKO.1 shRNA against mouse Myh9**	The Wistar Institute screening core	TRCN0000071504
**pLKO.1 shRNA against mouse Myh9**	The Wistar Institute screening core	TRCN0000071506
**pLKO.1 shRNA Scramble**	Addgene	136035
**pCMV-mCherry-MHC IIA**	Addgene	35687
**Flag-Myh9-Head**	This paper	N/A
**Flag-Myh9-Tail**	This paper	N/A
**mHif1α-TM**	Celest Simon Lab	N/A

Supplementary References

1. Jerby-Arnon, L. *et al.* A Cancer Cell Program Promotes T Cell Exclusion and Resistance to Checkpoint Blockade. *Cell* **175**, 984-997 e924 (2018).
