# Supplementary material for: Cell state dependent effects of Bmal1 on melanoma immunity and tumorigenicity
Source: Nat Commun. 2024 Jan 20;15:633. doi: 10.1038/s41467-024-44778-2 (PMC10799901; doi:10.1038/s41467-024-44778-2)
Supplement: Supplementary file 3 — Description of Additional Supplementary Files [file 41467_2024_44778_MOESM3_ESM.pdf]

Description of Additional Supplementary Files

Supplementary Data 1. RNAseq of B16 and aC3 for Fig.1

Supplementary Data 2. RNAseq of YUMM2.1 for Fig.2

Supplementary Data 3. ChIPseq_H3K4me3 for Fig.3

Supplementary Data 4. ChIPseq_H3K27me3 for Fig.3

Supplementary Data 5. Proteomics of TurboID system for Fig.4

Supplementary Data 6. RNAseq of YUMM2.1 cells with shRNA against Myh9 for Fig.6
