# Supplementary material for: Cell state dependent effects of Bmal1 on melanoma immunity and tumorigenicity
Source: Nat Commun. 2024 Jan 20;15:633. doi: 10.1038/s41467-024-44778-2 (PMC10799901; doi:10.1038/s41467-024-44778-2)
Supplement: Supplementary file 10 — Reporting Summary [file 41467_2024_44778_MOESM10_ESM.pdf]

nature portfolio

Corresponding author(s): Chi Dang

Last updated by author(s): Chi Dang, Xue Zhang

Reporting Summary

Nature Portfolio wishes to improve the reproducibility of the work that we publish. This form provides structure for consistency and transparency in reporting. For further information on Nature Portfolio policies, see our Editorial Policies and the Editorial Policy Checklist.

Please do not complete any field with "not applicable" or n/a. Refer to the help text for what text to use if an item is not relevant to your study.
For final submission: please carefully check your responses for accuracy; you will not be able to make changes later.

Statistics

For all statistical analyses, confirm that the following items are present in the figure legend, table legend, main text, or Methods section.

n/a	Confirmed	
☐	☑	The exact sample size (n) for each experimental group/condition, given as a discrete number and unit of measurement
☐	☑	A statement on whether measurements were taken from distinct samples or whether the same sample was measured repeatedly
☐	☑	The statistical test(s) used AND whether they are one- or two-sided<br>*Only common tests should be described solely by name; describe more complex techniques in the Methods section.*
☐	☑	A description of all covariates tested
☐	☑	A description of any assumptions or corrections, such as tests of normality and adjustment for multiple comparisons
☐	☑	A full description of the statistical parameters including central tendency (e.g. means) or other basic estimates (e.g. regression coefficient) AND variation (e.g. standard deviation) or associated estimates of uncertainty (e.g. confidence intervals)
☐	☑	For null hypothesis testing, the test statistic (e.g. F, t, r) with confidence intervals, effect sizes, degrees of freedom and P value noted<br>*Give P values as exact values whenever suitable.*
☑	☐	For Bayesian analysis, information on the choice of priors and Markov chain Monte Carlo settings
☑	☐	For hierarchical and complex designs, identification of the appropriate level for tests and full reporting of outcomes
☑	☐	Estimates of effect sizes (e.g. Cohen's d, Pearson's r), indicating how they were calculated

Our web collection on statistics for biologists contains articles on many of the points above.

Software and code

Policy information about availability of computer code

Data collection	Lumicycle 32/96; Odyssey CLx infrared imaging system;NIS-Elements;CyteFinder II;OMERO;Agilent TapeStation; Illumina Nextseq 500; Thermo Q Exactive Plus mass spectrometer; ECHO Revolve; VICTOR Multilable plate reader; FACS Symphony A3.
Data analysis	Image J; Ilastik; Lumicycle data analysis software; Graphpad Prism 9; R packages msigbdr, clusterprofiler and enrichplot; MaxQuant 1.6.3.3; HOMER; Bowtie2; RSEM v1.2.12; DESeq2; BaSic algorithm; MATLAB script (version 2019a); FlowJo; bigWigAverageOverBed; Biorender. Code used in this study is available at Zenodo (DOI: 10.5281/zenodo.10182504).

For manuscripts utilizing custom algorithms or software that are central to the research but not yet described in published literature, software must be made available to editors and reviewers. We strongly encourage code deposition in a community repository (e.g. GitHub). See the Nature Portfolio guidelines for submitting code & software for further information.

Data

Policy information about availability of data

All manuscripts must include a data availability statement. This statement should provide the following information, where applicable:

- Accession codes, unique identifiers, or web links for publicly available datasets
- A description of any restrictions on data availability
- For clinical datasets or third party data, please ensure that the statement adheres to our policy

All RNA-seq and ChIP-seq data analyzed in our study were deposited into GEO database (https://www.ncbi.nlm.nih.gov/geo/query/acc.cgi?acc=GSE202289) with accession number GSE202289. The mass spectrometry proteomics data have been deposited into the ProteomeXchange (https://www.proteomexchange.org/) repository with the accession number PXD037077. Public Single cell RNA-seq data from Single Cell Portal (Study: Melanoma immunotherapy resistance) were analyzed (https://singlecell.broadinstitute.org/single_cell/study/SCP109/melanoma-immunotherapy-resistance).
The remaining data are available in this Article, Supplementary Information and Supplementary Data File.
Source data are provided with this paper.

Research involving human participants, their data, or biological material

Policy information about studies with human participants or human data. See also policy information about sex, gender (identity/presentation), and sexual orientation and race, ethnicity and racism.

Reporting on sex and gender	No human participants in our study
Reporting on race, ethnicity, or other socially relevant groupings	No human participants in our study
Population characteristics	No human participants in our study
Recruitment	No human participants in our study
Ethics oversight	No human participants in our study

Note that full information on the approval of the study protocol must also be provided in the manuscript.

Field-specific reporting

Please select the one below that is the best fit for your research. If you are not sure, read the appropriate sections before making your selection.

☑ Life sciences ☐ Behavioural & social sciences ☐ Ecological, evolutionary & environmental sciences

For a reference copy of the document with all sections, see nature.com/documents/nr-reporting-summary-flat.pdf

Life sciences study design

All studies must disclose on these points even when the disclosure is negative.

Sample size	Sample size for animal experiments was estimated as per of the experimental design for approval by the institutional animal use committee.
Data exclusions	No data exclusions
Replication	Different experiments were in different replicates. Please refer to figure legend.
Randomization	The mice used in tumor growth measurement were randomized. For the experiments with anti-PD1 treatment, mice with matched tumor size were randomized into Isotype or anti-PD1 treatment group.
Blinding	No blinding was peformed in our animal study because the outcomes of our studies is unknown to us. Further, for some of the animal experiments, tumor measurements were performed by a laboratory blinded to the experimental design.

Behavioural & social sciences study design

All studies must disclose on these points even when the disclosure is negative.

Study description	Behavioural and social science is not relavant to our study
Research sample	Behavioural and social science is not relavant to our study
Sampling strategy	Behavioural and social science is not relavant to our study
Data collection	Behavioural and social science is not relavant to our study
Timing	Behavioural and social science is not relavant to our study
Data exclusions	Behavioural and social science is not relavant to our study
Non-participation	Behavioural and social science is not relavant to our study
Randomization	Behavioural and social science is not relavant to our study

Ecological, evolutionary & environmental sciences study design

All studies must disclose on these points even when the disclosure is negative.

Study description	No ecologial, evolutionary and environmental sciences study was involved in our study
Research sample	No ecologial, evolutionary and environmental sciences study was involved in our study
Sampling strategy	No ecologial, evolutionary and environmental sciences study was involved in our study
Data collection	No ecologial, evolutionary and environmental sciences study was involved in our study
Timing and spatial scale	No ecologial, evolutionary and environmental sciences study was involved in our study
Data exclusions	No ecologial, evolutionary and environmental sciences study was involved in our study
Reproducibility	No ecologial, evolutionary and environmental sciences study was involved in our study
Randomization	No ecologial, evolutionary and environmental sciences study was involved in our study
Blinding	No ecologial, evolutionary and environmental sciences study was involved in our study

Did the study involve field work? ☐ Yes ☑ No

Field work, collection and transport

Field conditions	No field work in our study
Location	No field work in our study
Access & import/export	No field work in our study
Disturbance	No field work in our study

Reporting for specific materials, systems and methods

We require information from authors about some types of materials, experimental systems and methods used in many studies. Here, indicate whether each material, system or method listed is relevant to your study. If you are not sure if a list item applies to your research, read the appropriate section before selecting a response.

Materials & experimental systems

n/a	Involved in the study	
☐	☑	Antibodies
☐	☑	Eukaryotic cell lines
☑	☐	Palaeontology and archaeology
☐	☑	Animals and other organisms
☑	☐	Clinical data
☑	☐	Dual use research of concern
☑	☐	Plants

Methods

n/a	Involved in the study	
☐	☑	ChIP-seq
☐	☑	Flow cytometry
☑	☐	MRI-based neuroimaging

Anti-BMAL1 (D2L7G) Cell Signaling Cat#: 14020; Anti-SOX9 (D8G8H) Cell Signaling Cat#: 82630; Anti-SOX10 (D5V9L) Cell Signaling Cat#: 89356; Anti-HIF1a (D1S7W) Cell Signaling Cat#: 36169; Anti-CLOCK (D45B10) Cell Signaling Cat#: 515

Antibodies

Antibodies used

Anti-BMAL1 (D2L7G) Cell Signaling Cat#: 14020; Anti-SOX9 (D8G8H) Cell Signaling Cat#: 82630; Anti-SOX10 (D5V9L) Cell Signaling Cat#: 89356; Anti-HIF1a (D1S7W) Cell Signaling Cat#: 36169;
Anti-CLOCK (D45B10) Cell Signaling Cat#: 5157; Anti-ATF4 (D4B8) Cell Signaling Cat#: 11815; Anti-Ki67 (D3B5) Cell Signaling Cat#: 9129; Anti-H3K4me3 (C42D8) Cell Signaling Cat#: 9751;
H3K27me3 (C36B11) Cell Signaling Cat#: 9733; Anti-H3K27Ac Cell signaling Cat#: 8173; Anti-Actn4 (D7U5A) Cell Signaling Cat#: 15145; Normal Rabbit IgG Cell Signaling Cat#: 2729;
Anti-MLK1 Millipore Sigma Cat#: HPA030782; Anti-MYH9 Proteintech Cat#: 11128-1-AP; Anti-Filamin A (EP2405Y) Abcam Cat#: ab76289; Anti-HA (12CA5) Millipore Sigma Cat#: 11583816001;
Anti-EZH2 Active Motif Cat#: 39933; Anti-α-Tubulin Mouse Mab (DM1A) Millipore Sigma Cat#: CP06; Anti-BMAL1 (B-1) Santa Cruz Cat#: sc365645; Anti-TBP Cell Signaling Cat#: 44059;
Anti-c-Jun Cell Signaling Cat#: 9165; Anti-phospho-c-Jun (ser73) Cell Signaling Cat#: 3270; Anti-Junb Cell Signaling Cat#: 3753; Anti-Jund Cell Signaling Cat#: 5000;
Anti-Atf5 Millipore Sigma Cat#: ABE2614; Anti-Atf6 Cell Signaling Cat#: 65880; Anti-Flag Cell Signaling Cat#: 14793; Anti-Flag M2 Millipore Sigma Cat#: F1804; InVivoMab anti-mouse PD1 (CD279) (RMP1-14) BioXCell Cat#: BE0146;
InVivoMAb rat IgG2a isotype control (2A3) BioXcell Cat#: BE0089; Goat anti-Rabbit IgG (H+L) highly Crossed-Adsorbed Secondary Ab, Alexa Fluor 680 Invitrogen Cat#: A-21109; Anti-mouse IgG(H+L) (DyLight 800 4X PEG conjugate) Cell Signaling Cat#: 5257;
FITC anti-mouse TCR β chain (H57-597) Biolegend Cat#: 109205; FITC anti-mouse CD19 (6D5) Biolegend Cat#: 115505; Brilliant Violet 785TM anti-mouse/human CD11b (M1/70) Biolegend Cat#: 101243;
APC/Cyanine7 anti-mouse Ly-6G (1A8) Biolegend Cat#: 127623; PerCP-CyTM 5.5 Rat Anti-Mouse Ly-6C (AL-21) BD Biosciences Cat#: 560525; PE Rat Anti-Mouse F4/80 (BM8) eBioscience Cat#: 12-4801-80;
APC Hamster Anti-Mouse CD11c (HL3) BD Biosciences Cat#: 561119; Brilliant Violet 605TM anti-mouse I-A/I-E (M5/114.15.2) Biolegend Cat#: 107639; Brilliant Violet 421TM anti-mouse CD103 (2E7) Biolegend Cat#: 121421;
PE/Cyanine7 anti-mouse CD206 (C068C2) Biolegend Cat#: 141720; Alexa Fluor 700 anti-mouse CD45 (30-F11) Biolegend Cat#: 103128; FITC anti-mouse Ly-6G (1A8) Biolegend Cat#: 127605;
FITC Hamster Anti-Mouse CD11c (HL3) BD Biosciences Cat#: 557400; Brilliant Violet 785TM anti-mouse CD3ε (145-2C11) Biolegend Cat#: 100355; PE Mouse Anti-Mouse NK1.1 (PK136) BD Biosciences Cat#: 557391;
PerCP-Cy5.5 Rat Anti-Mouse CD45R/B220 (RA3-6B2) BD Biosciences Cat#: 561101; APC-CyTM7 Rat Anti-Mouse CD8α (53-6.7) BD Biosciences Cat#: 557654;
Brilliant Violet 605TM anti-Mouse CD4 (GK1.5) Biolegend Cat# : 100451; Purified Rat Anti-Mouse CD16/CD32 (Mouse BD Fc BlockTM) (2.4G2) BD Biosciences 553142; Alexa Fluor 555 anti-SOX9 (EPR14335) Abcam Cat#: ab202516;
Alexa Fluor 647 anti-Sox10 (SP267) Abcam Cat#: ab270151; Goat anti-Rabbit IgG (H+L) highly Crossed-Adsorbed Secondary Ab, Alexa Fluor 647 Invitrogen Cat#: A-21245

Validation

Anti-BMAL1 (D2L7G) Cell Signaling Cat#: 14020 was validated in mouse brain tissue on the manufacture's website. It is noted on the manufacture's website that anti-SOX9 (D8G8H) Cell Signaling Cat#: 82630 can react with mouse species and it was validated by our data (see immunoblots);
Anti-SOX10 (D5V9L) Cell Signaling Cat#: 89356 was validated in human cells on the manufacture's website and was also validated by our data in both human and mouse samples (See immunoblots for Fig. 3g and h). This antibody was cited 36 times.
Anti-HIF1a (D1S7W) Cell Signaling Cat#: 36169 was validated in human samples on the manufacture's website and was cited 316 times, also, it was validated by our data (see immunoblots).
Anti-CLOCK (D45B10) Cell Signaling Cat#: 5157 was validated in mouse samples on the manufacture's website and was cited 37 times.
Anti-ATF4 (D4B8) Cell Signaling Cat#: 11815 was validated in human samples by the manufacture and is noted that it reacts with mouse species. It was cited 769 times.
Anti-Ki67 (D3B5) Cell Signaling Cat#: 9129 was validated by the manufacture in mouse brain and was cited 351 times.
Anti-H3K4me3 (C42D8) Cell Signaling Cat#: 9751 was validated by the manufacture in mouse samples and was cited 543 times.
Anti-H3K27me3 (C36B11) Cell Signaling Cat#: 9733 was validated by the manufacture in mouse sample and was cited 1183 times.
Anti-H3K27Ac Cell signaling Cat#: 8173 was validated by the manufacture in mouse sample and was cited 401 times.
Anti-Actn4 (D7U5A) Cell Signaling Cat#: 15145 was validated by the manufacture in mouse sample and was cited 8 times.
Anti-MLK1 Millipore Sigma Cat#: HPA030782, as a Prestige antibody, it is developed and validated by the Human Protein Atlas (HPA) project.
Anti-MYH9 Proteintech Cat#: 11128-1-AP was validated by the manufacture with mouse samples and was cited 27 times.
Anti-Filamin A (EP2405Y) Abcam Cat#: ab76289 was validated by the manufacture in human samples with CRISPR/Cas9 knockout, and it can react with mouse species. It was cited 36 times.
Anti-HA (12CA5) Millipore Sigma Cat#: 11583816001 was validated by the manufacture. Anti-EZH2 Active Motif Cat#: 39933 was validated by the manufacture.
Anti-α-Tubulin Mouse Mab (DM1A) Millipore Sigma Cat#: CP06 was validated by our immunoblots data.
Anti-BMAL1 (B-1) Santa Cruz Cat#: sc365645 was validated by the manufacture. Anti-TBP Cell Signaling Cat#: 44059 was validated by the manufacture and cited 41 times.
Anti-c-Jun Cell Signaling Cat#: 9165 was validated by the manufacture with mouse sample and cited 1045 times.
Anti-phospho-c-Jun (ser73) Cell Signaling Cat#: 3270 was validated by the manufacture with mouse sample and cited 383 times.
Anti-Junb Cell Signaling Cat#: 3753 was validated by the manufacture in different species and was cited 84 times. Our data validated it with mouse samples.
Anti-Jund Cell Signaling Cat#: 5000 was validated by the manufacture in different species and was cited 14 times. Its reactivity with mouse species was validated by our data.
Anti-Atf5 Millipore Sigma Cat#: ABE2614 was validated by the manufacture only in monkey samples and its reactivity to mouse sample was validated by our data.
Anti-Atf6 Cell Signaling Cat#: 65880 was validated by the manufacture in human sample and was cited 162 times. Its reactivity to mouse sample was validated by our data.
Anti-Flag Cell Signaling Cat#: 14793 was validated by the manufacture and cited 1073 times. Anti-Flag M2 Millipore Sigma Cat#: F1804 was validated by the manufacture.
FITC anti-mouse TCR β chain (H57-597) Biolegend Cat#: 109205, the reactivity of this antibody to mouse was verified by the manufacture and there are 55 citations for it.
The reactivity of FITC anti-mouse CD19 (6D5) Biolegend Cat#: 115505 to mouse was verified by the manufacture and there are 65 citations for this antibody.
The reactivity of Brilliant Violet 785TM anti-mouse/human CD11b (M1/70) Biolegend Cat#: 101243 to mouse was verified by the manufacture and there are 48 citations for this antibody.
The reactivity of APC/Cyanine7 anti-mouse Ly-6G (1A8) Biolegend Cat#: 127623 to mouse was verified by the manufacture and there are 107 citations for it.
The reactivity of PerCP-CyTM 5.5 Rat Anti-Mouse Ly-6C (AL-21) BD Biosciences Cat#: 560525 to mouse was verified by the manufacture and there are 7 citations for this antibody.
The reactivity of PE Rat Anti-Mouse F4/80 (BM8) eBioscience Cat#: 12-4801-80 to mouse was verified by the manufacture and there are 256 citations.
The reactivity of APC Hamster Anti-Mouse CD11c (HL3) BD Biosciences Cat#: 561119 to mouse was verified by the manufacture and there are 7 citations.
The reactivity of Brilliant Violet 605TM anti-mouse I-A/I-E (M5/114.15.2) Biolegend Cat#: 107639 to mouse was verified by the manufacture and there are 26 citations for it.
The reactivity of PE Mouse Anti-Mouse NK1.1 (PK136) BD Biosciences Cat#: 557391 to mouse was verified by the manufacture and there are 11 citations for it.
The reactivity of PerCP-Cy5.5 Rat Anti-Mouse CD45R/B220 (RA3-6B2) BD Biosciences Cat#: 561101 was verified by the manufacture and there are 20 citations.
The reactivity of APC-CyTM7 Rat Anti-Mouse CD8α (53-6.7) BD Biosciences Cat#: 557654 was verified by the manufacture and there are 27 citations.
The reactivity of Brilliant Violet 605TM anti-Mouse CD4 (GK1.5) Biolegend Cat#: 100451 was verified by the manufacture and there are 43 citations.

Eukaryotic cell lines

Policy information about cell lines and Sex and Gender in Research

Cell line source(s): YUMM2.1 and YUMM1.7 cell lines are from Ashani Weeraratna's lab. B16-F10 Bmal1 knockout clone and control clone are from Amita Sehgal's lab. B16-F10 parental and 293T cell line was purchased from ATCC. Human melanoma cell lines are from Meenhard Herlyn's lab. Other cell lines generated from YUMM2.1 and B16-F10 were made in our lab, including: YUMM2.1-EV, WT and dHLH, YUMM2.1 EV shNC, YUMM2.1 EV shMyh9, YUMM2.1 WT shNC, YUMM2.1 WT shMyh9, YUMM2.1 mHif1a, aC3 EV, aC3 mHif1a, aC3 WT, aC3 dHLH, B16 EV and B16-mHif1a, YUMM2.1 Tb, YUMM2.1 TbNLS, YUMM2.1 TbWT, and YUMM2.1 TbdHLH, all Bmal1 KO clones and control clones.

Authentication: We did not do the authentication for these cell lines.

Mycoplasma contamination: No mycoplasma contamination

Commonly misidentified lines (See ICLAC register): None of them is commonly misidentified lines

Palaeontology and Archaeology

Specimen provenance: Our study is not related to palaeontology or archaeology

Specimen deposition: Our study is not related to palaeontology or archaeology

Dating methods: Our study is not related to palaeontology or archaeology

☐ Tick this box to confirm that the raw and calibrated dates are available in the paper or in Supplementary Information.

Ethics oversight: Our study is not related to palaeontology or archaeology

Note that full information on the approval of the study protocol must also be provided in the manuscript.

Animals and other research organisms

Policy information about studies involving animals; ARRIVE guidelines recommended for reporting animal research, and Sex and Gender in Research

Laboratory animals: Seven to nine weeks old C57BL/6 mice, NSG mice were kept in 12 hrs/ 12hrs dark/light cycle. The ambient temperature is 68-72°F and humidity is 40-60

Wild animals: We did not use any wild animals

Reporting on sex: male

Field-collected samples: None field-collected sample was used in our study

Ethics oversight: Animal protocols were approved by Institutional Animal Care and Use Committee (IACUC) at Wistar Institute (Protocol number: 201189) and Animal Care and Use Committee (ACUC) at Johns Hopkins University School of Medicine (Protocol number: MO22M452).

Note that full information on the approval of the study protocol must also be provided in the manuscript.

Clinical data

Policy information about clinical studies

All manuscripts should comply with the ICMJE guidelines for publication of clinical research and a completed CONSORT checklist must be included with all submissions.

Clinical trial registration: No clinical studies were involved in our study

Study protocol: No clinical studies were involved in our study

Data collection: No clinical studies were involved in our study

Outcomes: No clinical studies were involved in our study

Dual use research of concern

Policy information about dual use research of concern

Hazards

Could the accidental, deliberate or reckless misuse of agents or technologies generated in the work, or the application of information presented in the manuscript, pose a threat to:

No	Yes	
☑	☐	Public health
☑	☐	National security
☑	☐	Crops and/or livestock
☑	☐	Ecosystems
☑	☐	Any other significant area

Experiments of concern

Does the work involve any of these experiments of concern:

No	Yes	
☑	☐	Demonstrate how to render a vaccine ineffective
☑	☐	Confer resistance to therapeutically useful antibiotics or antiviral agents
☑	☐	Enhance the virulence of a pathogen or render a nonpathogen virulent
☑	☐	Increase transmissibility of a pathogen
☑	☐	Alter the host range of a pathogen
☑	☐	Enable evasion of diagnostic/detection modalities
☑	☐	Enable the weaponization of a biological agent or toxin
☑	☐	Any other potentially harmful combination of experiments and agents

Plants

Seed stocks	We did not use any plants in our study
Novel plant genotypes	We did not use any plants in our study
Authentication	We did not use any plants in our study

ChIP-seq

Data deposition

☑ Confirm that both raw and final processed data have been deposited in a public database such as GEO.

☑ Confirm that you have deposited or provided access to graph files (e.g. BED files) for the called peaks.

Data access links<br>*May remain private before publication.*	https://www.ncbi.nlm.nih.gov/geo/query/acc.cgi?acc=GSE202289
Files in database submission	GSE202289_RAW.tar
Genome browser session<br>(e.g. UCSC)	UCSC

Methodology

Replicates	2
Sequencing depth	the sequencing reads number for each sample is more than 31,000,000
Antibodies	H3K4me3, H3K27me3 and H3K27 Ac, Rabbit IgG
Peak calling parameters	HOMER was used to call significant peaks using "-style histone" option
Data quality	With aligned ratio is larger than 93% for all samples, the unique percentage for all samples is great thatn 80%

Software | Bowtie and HOMER

Flow Cytometry

Plots

Confirm that:

☑ The axis labels state the marker and fluorochrome used (e.g. CD4-FITC).

☑ The axis scales are clearly visible. Include numbers along axes only for bottom left plot of group (a 'group' is an analysis of identical markers).

☑ All plots are contour plots with outliers or pseudocolor plots. The example for gating strategy in Supplementary Fig. 2i was shown in dot plots

☑ A numerical value for number of cells or percentage (with statistics) is provided.

Methodology

Sample preparation	Tumor tissues were dissociated into single cell suspension; cell lines were trypsinized into single cell suspension.
Instrument	gentleMACS Octo Dissociator with Heaters
Software	37C_m_TDK_1 with Heaters
Cell population abundance	YUMM2.1 tumors have high immune cell infiltration including CD4T, CD8, macrophages, MDSCs, DCs, NK and B cells
Gating strategy	Single cells were gated from whole cell population with FSC-A/FSC-H, then live cells were gated via live/dead dye Aqua. After that, CD45+ cells were gated for all immune cells, then the specific subsets of lymphocytes and myeloid cells were seperatly gated from the lymphocytes pannel and myeloid cells pannel. For more details, please see supplementary Fig. 2i

☑ Tick this box to confirm that a figure exemplifying the gating strategy is provided in the Supplementary Information.

Magnetic resonance imaging

Experimental design

Design type	We did not perform any magnetic resonance imaging
Design specifications	We did not perform any magnetic resonance imaging
Behavioral performance measures	We did not perform any magnetic resonance imaging
Imaging type(s)	We did not perform any magnetic resonance imaging
Field strength	We did not perform any magnetic resonance imaging
Sequence & imaging parameters	We did not perform any magnetic resonance imaging
Area of acquisition	We did not perform any magnetic resonance imaging

Diffusion MRI ☐ Used ☑ Not used

Preprocessing

Preprocessing software	We did not perform any magnetic resonance imaging
Normalization	We did not perform any magnetic resonance imaging
Normalization template	We did not perform any magnetic resonance imaging
Noise and artifact removal	We did not perform any magnetic resonance imaging
Volume censoring	We did not perform any magnetic resonance imaging

Statistical modeling & inference

Model type and settings	We did not perform any magnetic resonance imaging
Effect(s) tested	We did not perform any magnetic resonance imaging

Specify type of analysis: ☐ Whole brain ☐ ROI-based ☐ Both

Statistic type for inference (See Eklund et al. 2016): We did not perform any magnetic resonance imaging

Correction: We did not perform any magnetic resonance imaging

Models & analysis

n/a	Involved in the study
☑	☐ Functional and/or effective connectivity
☑	☐ Graph analysis
☑	☐ Multivariate modeling or predictive analysis

Functional and/or effective connectivity: We did not perform any magnetic resonance imaging

Graph analysis: We did not perform any magnetic resonance imaging

Multivariate modeling and predictive analysis: We did not perform any magnetic resonance imaging

This checklist template is licensed under a Creative Commons Attribution 4.0 International License, which permits use, sharing, adaptation, distribution and reproduction in any medium or format, as long as you give appropriate credit to the original author(s) and the source, provide a link to the Creative Commons license, and indicate if changes were made. The images or other third party material in this article are included in the article's Creative Commons license, unless indicated otherwise in a credit line to the material. If material is not included in the article's Creative Commons license and your intended use is not permitted by statutory regulation or exceeds the permitted use, you will need to obtain permission directly from the copyright holder. To view a copy of this license, visit http://creativecommons.org/licenses/by/4.0/
